# Competitive Immunoassay in a Microfluidic Biochip for In-Field Detection of Abscisic Acid in Grapes

**DOI:** 10.3390/bios14030123

**Published:** 2024-02-26

**Authors:** Cristiana Domingues, Rui Meirinho, Rodolfo G. Rodrigues, Ana Margarida Fortes, Virginia Chu, João Pedro Conde

**Affiliations:** 1 Instituto de Engenharia de Sistemas e Computadores–Microsistemas e Nanotecnologias (INESC-MN), Rua Alves Redol, 1000-029 Lisbon, Portugal; cdomingues@inesc-mn.pt (C.D.); rui.meirinho@inesc-mn.pt (R.M.); rodolfo.rodrigues@inesc-mn.pt (R.G.R.); vchu@inesc-mn.pt (V.C.); 2 Instituto de Biossistemas e Ciências Integrativas (BioISI), Faculdade de Ciências de Lisboa, Universidade de Lisboa, 1749-016 Lisbon, Portugal; amfortes@ciencias.ulisboa.pt; 3 Department of Bioengineering, Instituto Superior Técnico, Avenida Rovisco Pais, 1049-001 Lisbon, Portugal

**Keywords:** microfluidics, abscisic acid, competitive immunoassay, biosensing, agriculture

## Abstract

Viticulture and associated products are an important part of the economy in many countries. However, biotic and abiotic stresses impact negatively the production of grapes and wine. Climate change is in many aspects increasing both these stresses. Routine sample retrievals and analysis tend to be time-consuming and require expensive equipment and skilled personnel to operate. These challenges could be overcome through the development of a miniaturized analytic device for early detection of grapevine stresses in the field. Abscisic acid is involved in several plant processes, including the onset of fruit ripening and tolerance mechanisms against drought stress. This hormone can be detected through a competitive immunoassay and is found in plants in concentrations up to 10^−1^ mg/mL. A microfluidic platform is developed in this work which can detect a minimum of 10^−11^ mg/mL of abscisic acid in buffer. Grape samples were tested using the microfluidic system alongside benchmark techniques such as high-performance liquid chromatography. The microfluidic system could detect the increase to 10^−5^ mg/mL of abscisic acid present in real berry samples at the *veraison* stage of ripening.

## 1. Introduction

Our planet is facing a climate change crisis, due to factors such as burning fossil fuels, cutting down forests, and farming livestock, leading to an increase in the planet’s temperature [1]. This increase in temperature, in addition to the rise of seawater levels resulting from the melting of the polar ice caps, affects biodiversity and causes an increase in the spread of pests and pathogens. In this way, climate change critically threatens the natural ecosystem, causing floods, droughts, and heat stress for plants, where vines are no exception [2]. As stated in the 2019 statistical report from the International Organization of Vine and Wine (OVI), in 2018 there was a world production of 77.8 million tons of grapes, with 57% of these destined for winemaking, 36% for table grapes and 7% for raisins [3]. Vines are susceptible to climate change, as they need a specific set of conditions to be able to fully develop. Thus, it is necessary to prevent biotic stresses (pathogenic infections) and abiotic stresses (e.g., drought, heat, cold, salinity) [4,5,6]. Several phytohormone concentrations are altered under these stresses, such as jasmonic acid (JA), azelaic acid (AzA), and salicylic acid (SA) for infections, and abscisic acid (ABA) and indole-3-acetic acid (IAA) for drought stress [7,8,9,10].

In particular, ABA (molar mass: 264.32 g/mol [11]) is a small molecule that regulates several biological processes in plants, such as water use efficiency, tolerance to osmotic stress, and seed germination. ABA is mostly synthesized in the roots in response to drought stress and transported to the leaves, although the leaves are also capable of producing ABA. During a drought period, the ABA produced in the roots is transported to the leaves where the osmotic potential of the stomatal cells is rapidly altered, causing the stomata to close, reducing transpiration and preventing water loss from the leaves [12,13]. Thus, when under stress, this phytohormone tends to rise. In addition to this, ABA is also important in the process of development and onset of ripening of grape berries [14]. Its concentration increases during the *veraison* stage when the berries change colour due to anthocyanin accumulation [14]. The concentration of ABA varies depending on the grape variety, so a certain concentration of ABA in one type of grape may be considered normal, while in another it can be considered as a sign of stress [15]. Therefore, it is important to monitor the ABA concentration over time to establish a baseline to use its concentration to monitor the condition of the plant.

That said, with the method developed in this work it is possible to detect drought stress before starting to see physiological and morphological changes in the grape, such as changes in leaves (withering), or even leaf curling, or lifting of the stem [16,17]. It can also be used for monitoring the dynamics of pre-harvest and post-harvest fruit ripening in association with other markers [14].

In order to have continuously updated information on the state of the plant, it is essential to develop a fast, inexpensive, and easy-to-use device to diagnose biotic and abiotic stresses in the field (point-of-impact) to avoid delayed or unnecessary countermeasures, such as application of irrigations or fungicides. Typically, the biomarkers referred to above are found in concentrations ranging between 10^−5^ to 10^−1^ mg/mL, and the most commonly used detection method is High-Performance Liquid Chromatography (HPLC) [18]. HPLC is an analytical technique used to first separate and then detect the components present in a solution. However, this technique is not suitable for field testing due to its bulk and the need for skilled personnel to operate the equipment, relatively long analysis time, and high costs [19,20].

Microfluidics technology may address these limitations through the development of devices that can provide a portable, easy-to-use, and high-throughput solution to in-field testing. Compared to standard immunoassays (e.g., ELISA), assays made with microfluidic devices use smaller quantities of reagents and have faster reaction times due to smaller diffusion lengths [21,22]. These advantages of microfluidic devices meet all the important criteria of an immunoassay used for environmental analyses, clinical diagnoses, and biochemical studies [23]. Early application of microfluidics for hormone detection in grapes was developed by Brás et al., with an enzymatic assay to quantify AzA based on the inhibitory effect of AzA on the activity of the tyrosinase enzyme [24]. However, in this method, the detection of the biomarker is performed indirectly. Due to the complexity of the grape matrix, enzyme activity may be inhibited by a compound other than AzA, resulting in an erroneous result. Antibodies, compared to enzyme-base assays, are more suitable for biomarker detection, given that the target is captured directly and does not depend on any additional reaction that could result in a loss of selectivity. In this work, a novel competitive immunoassay capable of detecting ABA in the buffer in a microfluidic device was developed. This assay was also capable of detecting ABA in real berry samples, making use of a sample pre-treatment protocol also developed in this work. This platform was validated with measurements of ABA in real berry samples at the *veraison* stage that were also performed by HPLC.

## 2. Materials and Methods

### 2.1. Fabrication of the Microfluidic Devices

**Figure 1:**
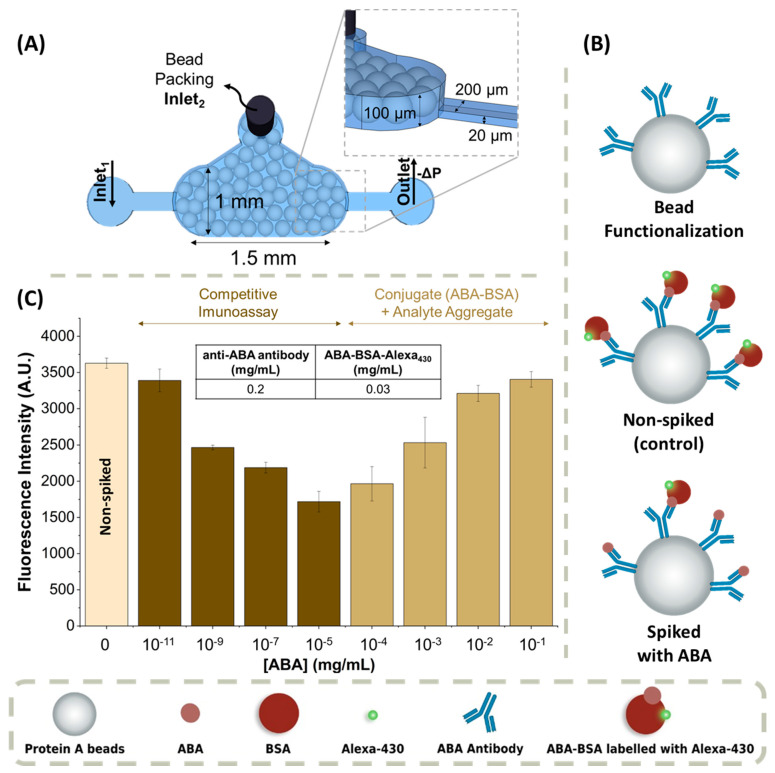
Competitive Immunoassay for ABA detection: (**A**) schematic of the microfluidic structures, packed with protein A microbeads for the detection of ABA; (**B**) schematic of the competitive fluorescence immunoassay: bead functionalization (the anti-ABA antibody is bound to the bead through the constant zone of the anti-ABA antibody, leaving the binding site available for binding to the target); control, non ABA-spiked assay (only the labelled ABA-BSA conjugate is present in the system, so only this ABA-BSA conjugate will bind to the anti-ABA antibody, resulting in the maximum signal in the absence of competition for the anti-ABA antibody sites); and ABA-spiked assay (both the labelled ABA-BSA conjugate and the free analyte are present in the system, so both will compete for the anti-ABA antibody binding sites: the higher the ABA concentration, the lower the fluorescence); (**C**) fluorescence response curve for different target ABA concentrations, ranging from 10^−11^ mg/mL to 10^−1^ mg/mL (n = 4). The excitation wavelength was 450–490 nm (blue). The error bars represent the ± standard deviation.

**Figure 2:**
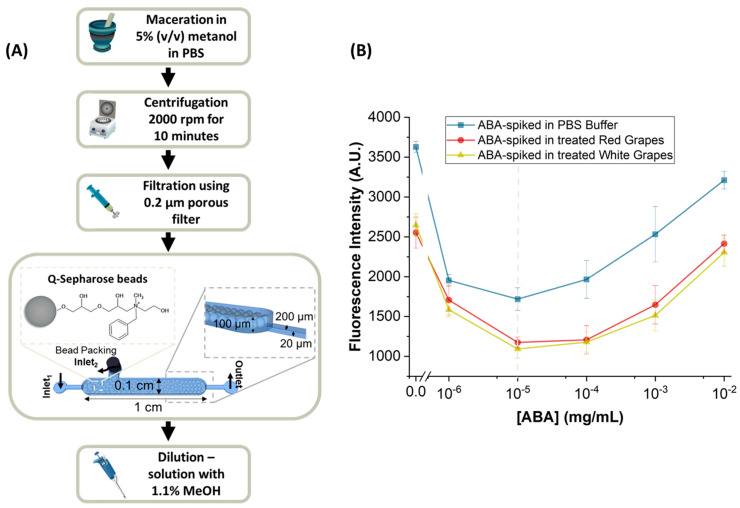
Sample treatment protocol and calibration curves for red and white treated table grapes: (**A**) overview of the steps carried out in the sample treatment: maceration of the sample; centrifugation (2000 rpm, 10 min); filtration (pore diameter: 0.2 µm); and bead cleaning step in a microfluidic channel (the columns used were 1 cm long, with a width of 0.1 cm, and a height of 100 µm, and, additionally, they had a smaller channel with 200 µm of width and a height of 20 µm designed to trap beads with diameters superior to 20 µm); (**B**) ABA detection competitive immunoassay performed in ABA-spiked and treated table grape samples, with ABA concentrations ranging from 10^−6^ at 10^−2^ mg/mL (n = 2). The excitation wavelength was 450–490 nm (blue). The error bars represent the ± standard deviation.

The microfluidic devices for ABA detection and sample treatment were fabricated with two different heights: (1) a channel with a height of 100 µm was used for bead packing, and (2) a channel with a height of 20 µm for the sample and reactant injection. The shallower channels allow trapping microbeads with diameters greater than 20 µm. The fabrication of the microfluidic devices containing immobilized microbeads is described elsewhere and briefly summarized below [25]. These devices were fabricated using a two-mask microfabrication process, each for a different height. A schematic drawing of the microfluidic devices is shown in Figure 1A and Figure 2A.

The photomasks were designed using Computer Assisted Design (CAD) software (AutoCAD 2022, Autodesk Inc., Mill Valley, CA, USA) and were fabricated in-house using direct-write photolithography (Heidelberg DWLII, Heidelberg Instruments Mikrotechnik, Heidelberg, Germany) after sputtering a 200 nm aluminium (Al) layer on a glass substrate using a Nordiko 7000 DC magnetron sputtering system (Nordiko Technical Services LTD, Hampshire, UK). Two different patterned Al masks were fabricated on glass substrates, one for each height of the final channel, to fabricate a two-level mould pattern using SU-8 2015 (Microchem, Newton, MA, USA) for the 20 μm layer and SU-8 50 (Microchem, Newton, MA, USA) for the 100 μm layer. The SU-8 photoresists were exposed to a UV light through the respective photomask, followed by a post-exposure bake and development step in propylene glycol methyl ether acetate (PGMEA, 99.5%, Sigma-Aldrich, St. Louis, MO, USA). After patterning the SU-8 features and a final hard bake step at 150 °C, for 15 min, the finished SU-8 mould was used to fabricate the standard process of replication of a polydimethylsiloxane (PDMS) structure, using Sylgard 184 silicon elastomer kit (Dow Corning, Midland, MI, USA). The PDMS was mixed at a ratio of 10 parts to 1 part curing agent, and poured on top of the mould. The PDMS was subsequently baked for 90 min at 70 °C, and then peeled from the mould. Holes were punched with an 18-gauge syringe (Instech Solomon, Plymouth Meeting, PA, USA) through the inlets and outlets of the structure. The devices containing the microfluidic structures were sealed against a 500 μm PDMS membrane, exposing both surfaces to an oxygen plasma (Harrick Plasma, Ithaca, NY, USA). In order to stabilize their hydrophobicity, the PDMS structures were stored for at least 24 h before being used [26]. Figure S1 in the Supplementary Materials shows the sequence of steps involved in the fabrication of the microfluidic devices used for trapping beads.

### 2.2. Competitive Immunoassay for ABA Detection

#### 2.2.1. Reagents

Protein A microbeads were obtained from Cytiva (Uppsala, Sweden). Phosphate Buffer Solution (PBS) 10×, casein (1% *w*/*v*), and Alexa Fluor^®^ (A430) NHS ester (succinimidyl ester) were obtained from Fisher Scientific (Waltham, MA, USA). Anti-ABA antibody (14.3 mg/mL) was obtained from Agrisera (Vännäs, Sweden). ABA-BSA conjugate (4 mg/mL) was obtained from Creative Diagnostics (Shirley, NY, USA). ABA (50 mg/mL), sodium bicarbonate buffer, dimethyl sulfoxide (DMSO), and methanol (MeOH) anhydrous 99.8% were obtained from Sigma-Aldrich (St. Louis, MO, USA).

#### 2.2.2. Functionalization of the Microbeads

The protein-A microbeads (diameter ~90 µm) were first incubated with the anti-ABA antibody solution. The incubation was performed by adding 3 µL of bead stock to 19 µL of anti-ABA antibody at 0.2 mg/mL. After 60 min of incubation, 110 µL of PBS was added to homogenise the bead suspension. These 3 µL of the bead stock always have approximately the same number of beads. If there is a different number of beads in different incubations, the anti-ABA antibody concentration on the beads will vary, and therefore a different number of binding sites will be available which will be a source of irreproducibility for the assay.

#### 2.2.3. Packing the Biomarker Detection Chamber

To insert the beads into the microfluidic structure, a negative pressure was applied at the both the outlet and at inlet_1_ with a syringe pump (NE-4000, New Era Pumps, Farmingdale, NY, USA). The channel was first filled with PBS 1× buffer and a pipette tip containing 20 µL of bead solution was inserted in the bead chamber inlet_2_ as shown in Figure 1A. The negative pressure produced a flow rate of approximately 6 µL/min in the channel, resulting in the packing of the beads inside the detection chamber. Subsequently, the microfluidic structure was washed at a flow rate of 16 µL/min with PBS 1×, and the bead chamber inlet_2_ was sealed using a 20-gauge metal plug. The bead-packed structure was stored in a container filled with deionised water (DI) and refrigerated overnight to remove any air bubbles formed during the microbeads insertion process.

#### 2.2.4. ABA-BSA Conjugate Labelling

To prepare fluorescently labelled ABA-BSA conjugate, 20 µL of ABA-BSA conjugate was diluted in 480 µL of 0.1 M sodium bicarbonate buffer and then conjugated with 2 µL of the amine-reactive dye Alexa Fluor^®^ (A430) NHS ester (succinimidyl ester) previously dissolved in DMSO at 10 mg/mL. This mixture between ABA-BSA conjugate and Alexa430 was incubated in the dark for 60 min at room temperature. To remove the excess non-conjugated dye, a series of washing steps were performed, using a 10 kDa Amicon Ultra-0.5 centrifugal filter unit (Merck, Alameda Fernão Lopes, Algés, Portugal). The incubated solution was added to the Amicon tube, and centrifuged at 14,000 times gravity (× *g*) for 10 min. After centrifugation was complete, the permeate at the bottom of the tube was discarded, and 500 µL of PBS 1× was added to initiate the washing process and the solution was again centrifuged at 14,000× *g* for 10 min. This latter step was performed by repeated cycles until the permeate became transparent. Then, the labelled ABA-BSA conjugate solution was collected by reverse centrifugation at 2000× *g* for 2 min and the remaining volume was measured and made up to the initial volume with PBS 1×, to set the concentration.

#### 2.2.5. ABA Detection Immunoassay

The microcolumn was packed with functionalized microbeads, as described in Section 2.2.2. To minimize non-specific interactions with both the microchannel PDMS surface and the agarose beads, a solution of 0.1% casein (*w*/*v*) was pumped at a flow rate of 1 µL/min for 10 min. Then, the labelled ABA-BSA conjugate (0.03 mg/mL) together with a given concentration of the analyte in the calibration assays or the prepared sample were pumped through the channel at a flow rate of 1 µL/min flow rate for 5 min (while keeping a constant percentage of MeOH of 1%), followed by a washing step with PBS 1× with a flow rate of 5 µL/min for 10 min, to remove any unbound ABA-BSA conjugate and analyte.

For the control assay, which was not spiked with ABA, only the labelled ABA-BSA conjugate was introduced into the microchannel. In the case of an ABA spiked assay or in an assay with prepared grapes or leaf samples (see Section 2.3), the labelled ABA-BSA conjugate flowed together with the sample to be measured.

#### 2.2.6. Image Acquisition and Processing

The acquisition signal from the experimental assay was performed using a fluorescence microscope (Leica DMLM) equipped with a digital camera (DFC300FX) and a CoolLED lamp (pE-300lite) as an excitation light source, coupled to an I3 filter cube with a band-pass for excitation of 450–490 nm (blue) and a long pass for emission at 515 nm (green). The images acquired were analysed using ImageJ software from the National Institutes of Health, USA [27], where only the green channel was appraised. The value obtained through this green channel results from the subtraction between an inner area of the channel (signal), and an equal-sized area outside the channel (background). All fluorescence micrographs used for ABA quantification were acquired using exposure time of 2 s, and 1 × gain.

### 2.3. Sample Collection and Treatment for ABA Detection

To establish the sensitivity of the ABA detection assay, samples of real table grapes, white (Beauty Sweet Gold) and red (Red Globe), were bought at the supermarket and kept at −80 °C until use. These grapes have a low ABA content compared to grapes that are in the *veraison* stage.

To validate the ABA detection method, berries from the Touriga Nacional cultivar were collected from a local vineyard at the *veraison* stage, frozen in liquid nitrogen, transported to the laboratory in dry ice, and kept at −80 °C until use.

The protocol developed for sample treatment consisted of first macerating the sample in a mortar with a 5% MeOH solution diluted in PBS 1×, at a ratio of 1 g of sample to 1 mL of solution, for 8 min. This first step had the purpose of dissolving and extracting the biomarkers present in the plant tissues. Afterwards, the samples were centrifuged at 2000 rpm for 10 min, and the supernatants were collected and then filtered using a Nylon syringe filter with a 0.2 µm pore diameter. In the next step, to remove any remaining contaminants that did not come out during filtration, such as debris, these supernatants were cleaned using Q-Sepharose beads packed in microfluidic channels. The supernatant was pumped through the channel with Q-Sepharose beads at a flow rate of 5 µL/min for 10 min. A final dilution was performed to reach a constant composition of 1.1% MeOH in all samples, given that during preliminary microfluidic experiments it was verified that high percentages of MeOH caused interference with the molecular recognition assay.

#### Packing of the Sample Treatment Chamber

To insert the Q-Sepharose microbeads (diameter ~90 µm) from Cytiva (Uppsala, Sweden) into the microfluidic structure, a negative pressure was applied at the outlet using a syringe pump. The channel was first filled with PBS 1× buffer and a pipette tip containing 30 µL of bead solution (prepared at a ratio of 1:10 with its bead stock in 20% ethanol) was inserted in the chamber inlet_2_ (Figure 2A). The negative pressure produced a flow rate of approximately 12 µL/min into the channel, resulting in the packing of the beads inside the sample treatment chamber. Subsequently, the microfluidic structures were washed at 22 µL/min with PBS 1×, and the bead chamber inlet_2_ (Figure 2A) was sealed using a 20-gauge metal plug.

### 2.4. Reverse-Phase HPLC Detection of ABA

The concentration of ABA in the grape samples was confirmed using a Hitachi LaChrom HPLC system. The HPLC system consists of two pumps (L-7100), a UV-detector (L-7400), a programmable autosampler (L-7250), and an interface (Hitachi D-7000) to connect the system to a computer. The column used was a core-shell organo-silica LC column from Kinetex (5 µm EVO C18 100 Å, 250 × 4.6 mm) with UV detection at 206 nm. The analysis was performed in isocratic mode with sodium phosphate buffer (50 mM, pH 3.55, Sigma-Aldrich, St. Louis, MO, USA) and acetonitrile (VWR, Carnaxide, Portugal) as the mobile phase (75:25 *v*/*v*), an injection volume of 25 µL, and a flowrate of 1.2 mL/min, following the method described in Brás et al. [24].

## 3. Results and Discussion

The main objective of this work was the development of a method capable of detecting ABA in grapes using a microfluidic platform. In this work, ABA was used as a biomarker for the onset of grape ripening (berry colour transition), i.e., the *veraison* stage. A competitive immunoassay was designed consisting of the immobilization of the anti-ABA antibody on Protein A microbeads which are mechanically trapped in the microfluidic channel (Figure 1A), followed by the optical detection of ABA in a competition between the ABA in the sample and a labelled ABA-BSA conjugate. This strategy is schematically summarized in Figure 1B. In this assay, in the absence of ABA, the fluorescence signal reaches its maximum, because there is no free analyte present to compete with the anti-ABA antibody capture sites. However, in the presence of ABA, the free analyte will compete with labelled ABA-BSA conjugate in solution for the binding sites of the anti-ABA antibody, and, as a consequence, the higher the concentration of the free analyte, the lower the fluorescence signal.

The use of microbeads in the microfluidic structure increases the surface area for probe immobilization, leading to an increase in the sensitivity of detection. Additionally, it decreases the diffusion length required for capture, and therefore the duration of the assay. Given that the incubation of the anti-ABA antibody and the microbeads is performed off-chip, and then the functionalized beads are packed in the microfluidic channels, it is necessary to ensure that the number of microbeads is approximately the same in each assay. This is necessary to maintain approximately constant the number of antibodies available, so that the conditions for the competition between the analyte and the ABA-BSA conjugate for the binding sites is kept constant.

### 3.1. ABA-Spiked in PBS 1× Buffer

The first step in developing the assay was to determine the concentrations of ABA-BSA conjugate and anti-ABA antibody to be used to obtain a competitive immunoassay. Figure S2 in Supplementary Materials shows a summary of the optimization steps. The anti-ABA antibody concentration cannot be too high because if there are too many binding sites, there will be no competition between the free analyte and the ABA-BSA conjugate, and both will bind to the anti-ABA antibody. The chosen ABA-BSA conjugate concentration considers the anti-ABA antibody concentration used to functionalize the beads. It must always be lower than the antibody concentration, because otherwise, the ABA-BSA conjugate would prevail over the analyte, resulting in no competition. After optimization, the chosen concentrations of anti-ABA antibody and ABA-BSA conjugate were 0.2 mg/mL and 0.03 mg/mL, respectively.

After determining the concentration of ABA-BSA conjugate and anti-ABA antibody, the dependence of the fluorescence signal with ABA concentration in PBS 1× buffer was measured and is shown in Figure 1C. To perform this experiment, successive dilutions of a solution with 50 mg/mL of ABA were measured to determine the lowest detectable ABA concentration using the microfluidic ABA detection immunoassay.

Figure 1C shows that the fluorescence dependence of ABA concentration yields a V-shaped graph. Between 10^−11^ and 10^−5^ mg/mL, the assay behaves as a typical competitive immunoassay, with the fluorescence decreasing as the concentration of ABA increases. However, for higher concentrations of analyte, between 10^−4^ and 10^−1^ mg/mL, the behaviour changes, and the fluorescence signal increases with increasing concentration of analyte, suggesting that there is aggregate formation between the ABA-BSA conjugate and the free analyte in solution. Figure S3 in Supplementary Materials shows a more detailed schematic diagram of the proposed formation of aggregates. This shape will have an impact on the measurement of real samples, as is discussed in Section 3.3.

The selectivity of this detection method was confirmed using a different analyte, SA, which, for concentrations around 10^−5^–10^−4^ mg/mL, showed no decrease in fluorescence with respect to the control, which was the non-ABA spiked assay.

### 3.2. ABA-Spiked in Treated Table Grape Samples

Having established the sensitivity of the ABA detection assay in model conditions (i.e., using ABA in PBS 1×), the next step was to establish the sensitivity of the ABA detection assay in real table grape samples. For this purpose, a sample treatment protocol was developed as shown schematically in Figure 2A (and described in detail in Section 2.3).

The role of the step of bead cleaning is removing any larger particles that escaped the step of filtration and that interfered with the analysis.

Using the protocol developed on table grapes, we obtained the curves shown in Figure 2B for the dependence of the fluorescence on the ABA concentration spiked in the grape samples. Table grapes were used in this study as they are easier to acquire. Both the curve obtained for the treated white grapes and for the treated red grapes match the curve obtained using PBS 1× buffer, confirming that the sample treatment protocol is efficient in removing the majority of the interferents from the grape samples. The decrease in fluorescence observed in assays with the real sample compared to the buffer is attributed to residual interferents.

Having developed a method capable of detecting ABA in PBS 1× buffer, as well as a sample treatment protocol capable of removing possible interferents in grapes, the next step is detecting ABA in wine grapes, under conditions in which a natural increase in ABA is expected.

### 3.3. ABA in Real Wine Grape Samples

**Figure 3:**
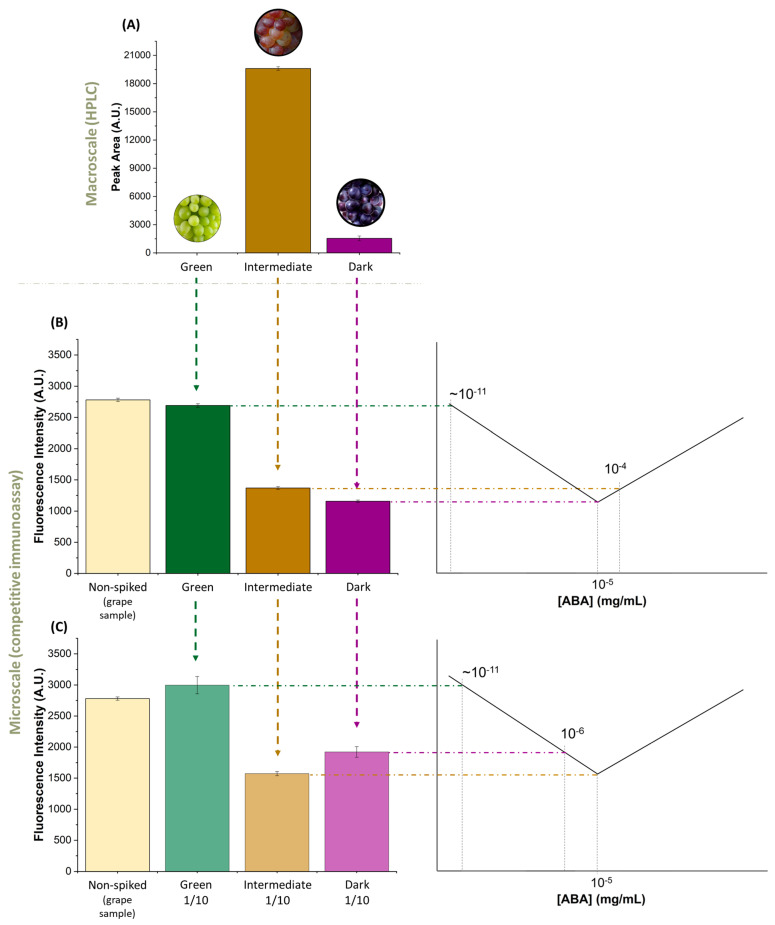
ABA detection method validation in real grape samples. The three tested samples, dark, intermediate, and green grapes, were selected from a grape bunch undergoing the veraison stage: (**A**) ABA concentration obtained from HPLC, where the peak area is directly proportional to the ABA concentration for each the tested samples; (**B**) ABA concentration obtained using microfluidic competitive immunoassay for ABA detection after the sample processing step; (**C**) microfluidic ABA detection competitive immunoassay performed in 10× diluted validation samples. The fluorescence intensity value of non-ABA spiked assay (table grape sample) was taken from Figure 2B, by averaging the fluorescence intensity of the non-ABA spiked of the treated red grapes with the fluorescence intensity non-ABA spiked of the treated white grapes. The error bars represent the ± standard deviation.

The grape samples chosen to validate the ABA detection method are those undergoing the *veraison* stage, which corresponds to the phase in which the berries are in a colour transition. During this phase, it is known that there is an increase in the concentration of ABA in the grape [15]. During the *veraison* stage, in the same cluster of grapes, some berries present a green colour, some dark, and others in between, revealing high heterogeneity in ripening. Samples were picked belonging to the three different stages, green, intermediate, and dark, as shown in the photos in Figure 3. The samples for “dark” and “green” grapes correspond to those that clearly show red and green colour, respectively. The “intermediate” grapes refer to samples that are exactly in the colour transition, where the berries have both colours. These samples were of the Touriga Nacional grape variety.

Before analysing the sample, the grapes were treated using the sample cleaning protocol mentioned in Section 2.3.

Using HPLC, it is possible to verify in Figure 3A that the ABA concentration is higher when the berry is exactly at the colour transition, as expected. Figure S4 in Supplementary Materials shows how the ABA concentration was estimated using HPLC. Thus, intermediate and dark grape samples present 5 × 10^−4^ mg/mL and 4 × 10^−5^ mg/mL of ABA concentration, respectively. The ABA concentration in the green grapes could not be detected by HPLC and is estimated to be around 10^−11^ mg/mL.

The results of the microfluidic assay for ABA detection, shown in Figure 3B, agree with the results obtained by HPLC. It should be noted that, as the detection method is competitive, the absence of ABA in the sample results in an increase in fluorescence signal (as can be seen in the green sample). The intermediate and dark grape samples, which have ABA concentrations from HPLC in the 10^−4^ mg/mL and 10^−5^ mg/mL range, respectively, follow the curve shown in Figure 2B, where the fluorescence signal in a sample with 10^−4^ mg/mL ABA is higher than the fluorescence signal in of a sample with 10^−5^ mg/mL ABA. Nevertheless, this conclusion is possible since the ABA concentrations were known from the HPLC measurements. To clarify the ambiguity in ABA concentration due to the V-shape of the curve in Figure 2B, the same processed grape samples used to validate the assay were diluted 10× and remeasured using the microfluidic system. These results are shown in Figure 3C. Figure 3C shows that the intermediate and dark grape samples, when diluted 10×, have ABA concentrations in the 10^−5^ mg/mL and 10^−6^ mg/mL range, respectively, and in this way, the intermediate sample now has a lower fluorescence signal than the dark sample. Furthermore, the signal in the green grape sample remains at approximately the same value after dilution, as expected, presenting a value close to that of non-ABA spiked table grapes (Figure 2B), having an ABA concentration in the 10^−11^ mg/mL range. In this way, successive dilutions can be performed if there is an ambiguity in the centration of ABA present in the sample.

Finally, it is important to mention that the concentrations of ABA present in the different *veraison* stage samples are effectively 5× higher than those shown in Figure 3 and mentioned in the discussion above because the final step of the sample treatment protocol is a 5× dilution. This way, the estimated ABA concentrations for the intermediate, dark, and green grape samples during the *veraison* stage are 2.5 × 10^−3^ mg/mL, 2 × 10^−4^ mg/mL, and 10^−11^ mg/mL, respectively.

The determination of the ABA concentration described above has the limitation that the calibration curve used (Figure 1) was not obtained with the same matrix as the real grape samples used (Figure 3). Although Figure 2 suggests that the effect of the matrix, after sample treatment, does not qualitatively alter the calibration, it would be more accurate, in critical practical applications, to perform a calibration of the ABA concentration in a matrix as close as possible to that of the sample under study.

## 4. Conclusions

A microfluidic biosensing platform capable of detecting an onset of ripening biomarker—ABA—was proposed, demonstrated, and validated. ABA, in addition to being representative of the onset of fruit ripening, is also a biomarker of drought stress; thus, the platform developed in this work can be used to monitor ABA concentration over time in a continuous manner, and thus control the water level in the plant, and experiments are under way to demonstrate this capability. A competitive immunoassay was developed, in which the anti-ABA antibody is functionalized on the surface of microbeads trapped in the microfluidic device, allowing for the detection of ABA using a labelled ABA-BSA conjugate. It was possible to detect ABA not only in buffer but also in real grape samples (both spiked and intrinsic), in concentrations ranging from 10^−11^ to 10^−1^ mg/mL, making this method more sensitive than any other ABA detection method reported in the literature [24,28]. Using HPLC, only ABA concentrations above 10^−5^ mg/mL could be detected.

The integration of this microfluidic biosensor, and additional biosensors for other relevant plant hormones, can potentially allow point-of-use, real-time control of grape biotic and abiotic stresses and onset of ripening, and improve timings of pesticide application and irrigation. It is noteworthy that ABA also regulates the same processes in other plants, such as strawberries and tomatoes, so this platform has the potential to be used in other plants than vines both during growth and also during processing, transportation, and storage.

## Data Availability

Data is contained within the article and Supplementary Materials.

## Supplementary Materials

The following supporting information can be downloaded at: https://www.mdpi.com/article/10.3390/bios14030123/s1, Figure S1: Sequence of steps involved in the fabrication of the (A) aluminum hard masks; (B) SU-8 negative photoresist mold; and (C) PDMS structures used for trapping beads. Figure S2: A selection of the results of optimization assays of the concentration of ABA-BSA conjugate to be used in the competitive immunoassay. Figure S3: Proposed mechanisms of ABA detection at different ABA concentrations: (A) schematic of the competitive fluorescence immunoassay, with the respective fluorescence micrograph, present at low ABA concentrations; (B) schematic of the aggregation between the ABA-BSA conjugate and the analyte, with the respective fluorescence micrograph, highlighting the presence of aggregates, present at high ABA concentrations. Figure S4: Calibration curve for different concentrations of ABA using in HPLC.
