# Supplementary material for: Competitive Immunoassay in a Microfluidic Biochip for In-Field Detection of Abscisic Acid in Grapes
Source: Biosensors (Basel). 2024 Feb 26;14(3):123. doi: 10.3390/bios14030123 (PMC10968099; doi:10.3390/bios14030123)
Supplement: Supplementary file 1 [file biosensors-14-00123-s001.zip › biosensors-2883823-supplementary.pdf]

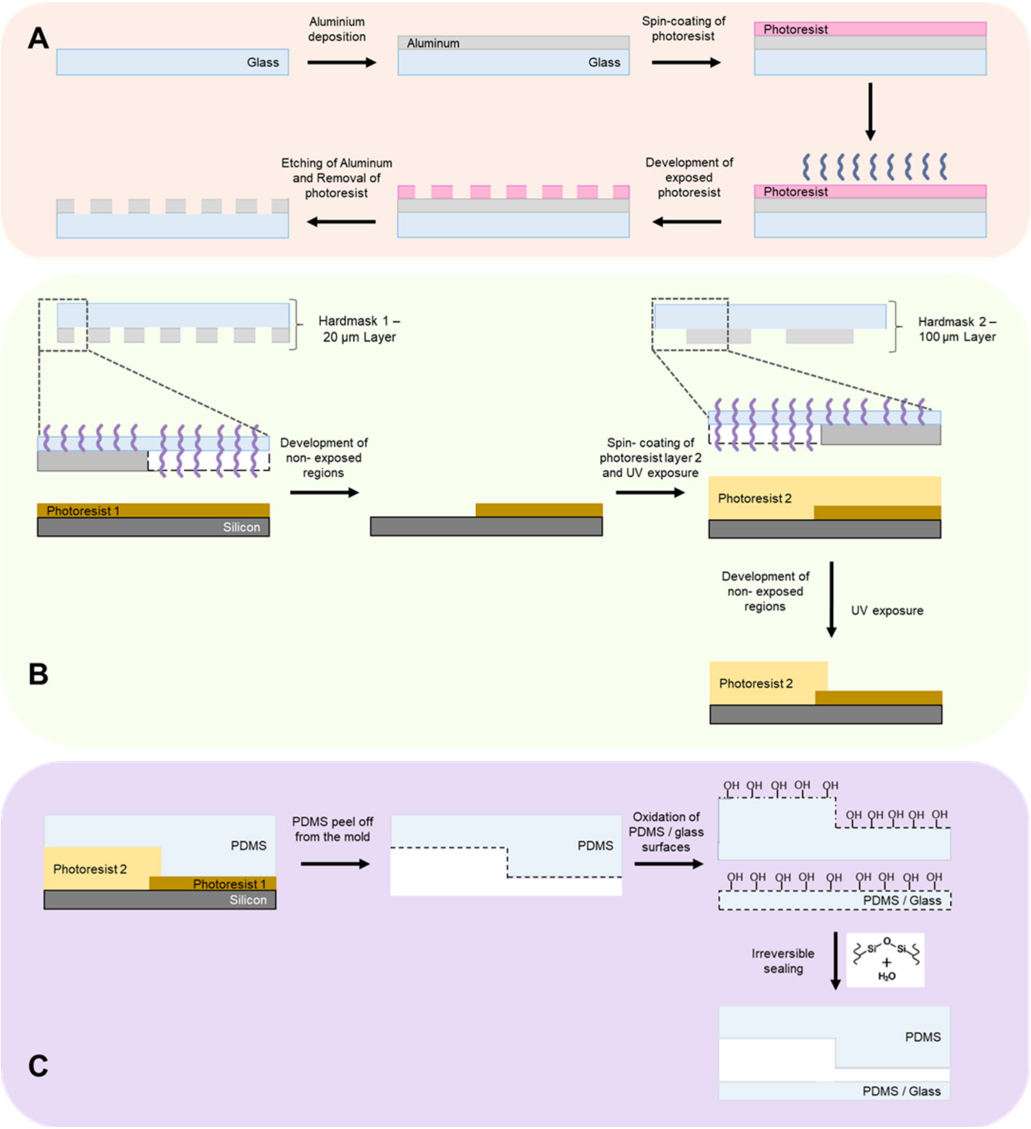


**Figure S1.** Sequence of steps involved in the fabrication of the **(A)** aluminum hard masks; **(B)** SU-8 negative photoresist mold; and **(C)** PDMS structures used for trapping beads.

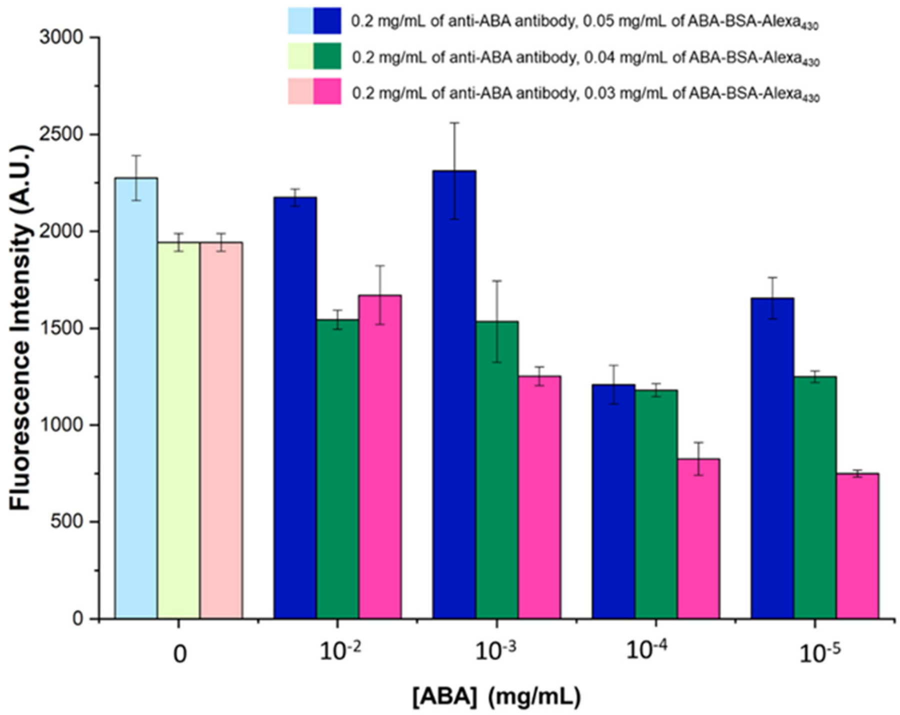


**Figure S2.** A selection of the results of optimization assays of the concentration of ABA-BSA conjugate to be used in the competitive immunoassay.

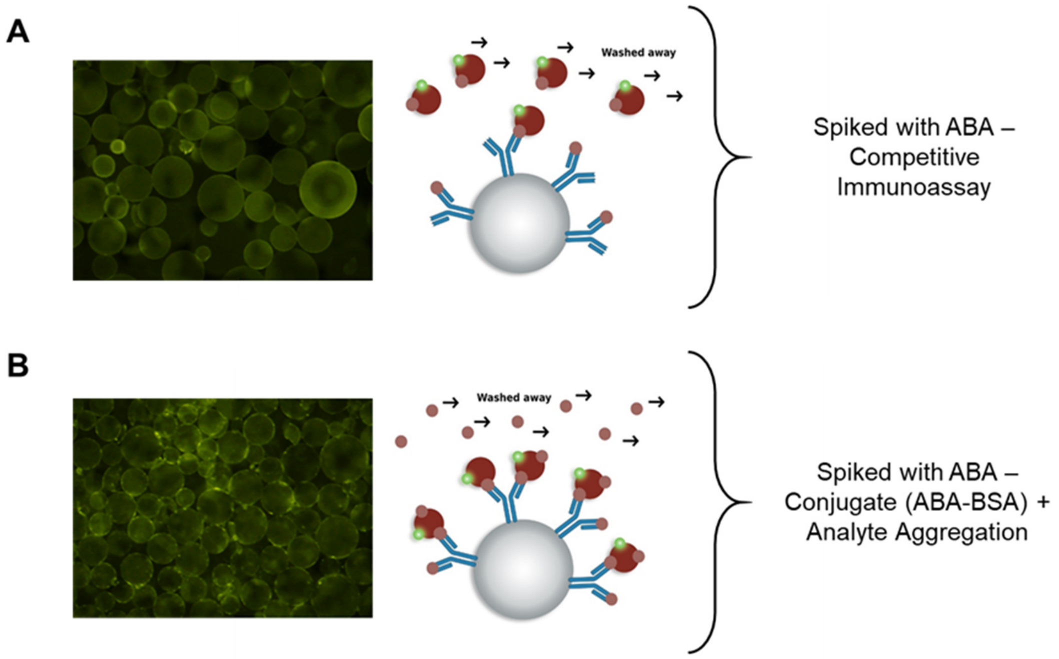


**Figure S3.** Proposed mechanisms of ABA detection at different ABA concentrations: (A) schematic of the competitive fluorescence immunoassay, with the respective fluorescence micrograph, present at low ABA concentrations; (B) schematic of the aggregation between the ABA-BSA conjugate and the analyte, with the respective fluorescence micrograph, highlighting the presence of aggregates, present at high ABA concentrations.

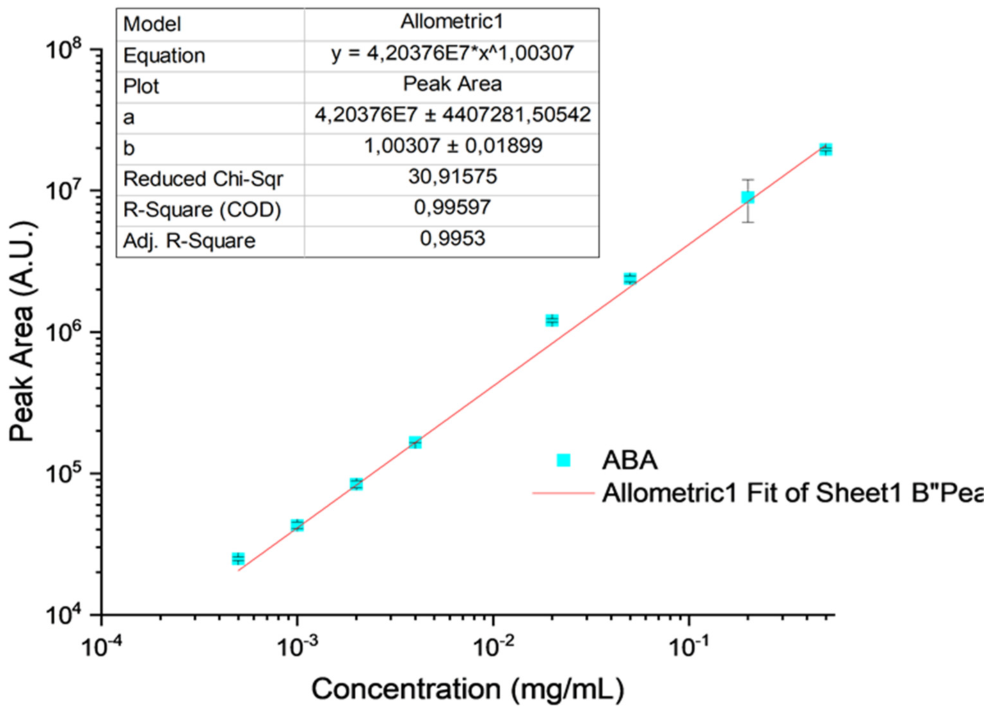


**Figure S4.** Calibration curve for different concentrations of ABA using in HPLC.
